# Proteinaceous Transformers: Structural and Functional Variability of Human sHsps

**DOI:** 10.3390/ijms21155448

**Published:** 2020-07-30

**Authors:** Mareike Riedl, Annika Strauch, Dragana A.M. Catici, Martin Haslbeck

**Affiliations:** Department Chemie, Technische Universität München, Lichtenbergstrasse 4, 85748 Garching, Germany; mareike.riedl@tum.de (M.R.); annika.strauch@tum.de (A.S.); dragana.a.m.catici@tum.de (D.A.M.C.)

**Keywords:** sHsp, α-crystallin, protein folding, protein assembly, eye lens

## Abstract

The proteostasis network allows organisms to support and regulate the life cycle of proteins. Especially regarding stress, molecular chaperones represent the main players within this network. Small heat shock proteins (sHsps) are a diverse family of ATP-independent molecular chaperones acting as the first line of defense in many stress situations. Thereby, the promiscuous interaction of sHsps with substrate proteins results in complexes from which the substrates can be refolded by ATP-dependent chaperones. Particularly in vertebrates, sHsps are linked to a broad variety of diseases and are needed to maintain the refractive index of the eye lens. A striking key characteristic of sHsps is their existence in ensembles of oligomers with varying numbers of subunits. The respective dynamics of these molecules allow the exchange of subunits and the formation of hetero-oligomers. Additionally, these dynamics are closely linked to the chaperone activity of sHsps. In current models a shift in the equilibrium of the sHsp ensemble allows regulation of the chaperone activity, whereby smaller oligomers are commonly the more active species. Different triggers reversibly change the oligomer equilibrium and regulate the activity of sHsps. However, a finite availability of high-resolution structures of sHsps still limits a detailed mechanistic understanding of their dynamics and the correlating recognition of substrate proteins. Here we summarize recent advances in understanding the structural and functional relationships of human sHsps with a focus on the eye-lens αA- and αB-crystallins.

## 1. Introduction

In cells various conditions can lead to the destabilization of proteins. Especially under stress conditions like heat, pH change, ultraviolet radiation, oxidative stress, the presence of heavy metals or other toxic substances, proteins tend to partially unfold and form aggregates [1,2]. In order to sustain cellular functions, organisms react to such stressors with the so-called heat stress response, which includes an increased expression of molecular chaperones. Small heat shock proteins (sHsps) are one family of molecular chaperones. They typically act within the first line of defense as they prevent proteins from irreversible aggregation in an ATP-independent fashion [3,4,5]. By binding the unfolding intermediates, they can keep unfolded substrate proteins in solution, which is often described as “holdase” function [6,7]. Under more severe stress conditions, sHsps even tend to co-precipitate with their non-native substrates in aggregate-like assemblies [4,8,9]. By overcoming the acute stress phase, substrate proteins can be recovered from these assemblies by ATP-dependent chaperones to be refolded or degraded [4,10]. Of note: in contrast to aggregation process in the absence of sHsps, the presence of sHsps enables efficient recovery of the non-native proteins [11,12,13,14].

**Figure 1:**
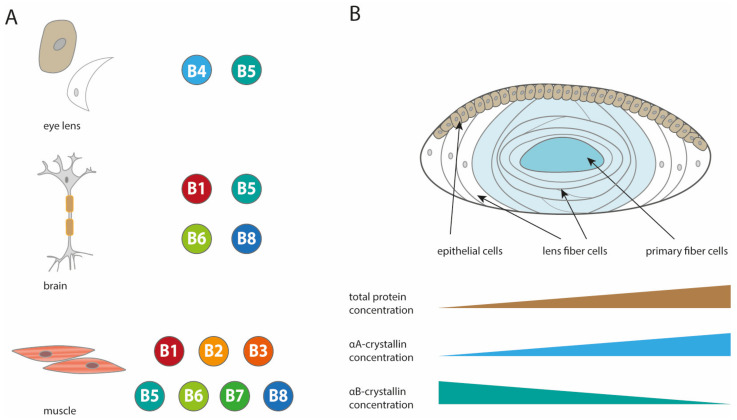
Expression of small heat shock proteins (sHsps) in the eye lens and other tissues. (**A**) Examples of differences in the expression of sHsps (at abundant levels) are shown for a selection of three tissues (eye lens, brain, muscle) [25,54,55]. (**B**) Simplified schematic model of the eye lens, epithelial cells, lens fiber cells (organelle free cells in light blue) and primary fiber cells [56]. Total protein concentration increases within the lens eyes. αA- and αB-crystallin concentration increase or decrease, respectively, during differentiation of bovine lens cells [57].

The sHsp family is characterized by its conserved α-crystallin domain (ACD) flanked by a variable, hydrophobic N-terminal region (NTR) and a shorter, variable, and polar C-terminal region (CTR) [3,15,16,17,18]. sHsps are the least conserved family of molecular chaperones as only the ACD represents a stringent recognition domain with a relative high homology across different family members. Interestingly, the ACD evolved independently of its flanking regions [19]. A further common feature of the sHsp family is their relatively small molecular weight, ranging between 12 and 42 kDa, combined with their ability to form large oligomeric complexes which, in most cases, contain ≥12 subunits [5,16,17,18]. In humans, ten ACD containing sHsps are known (HspB1–B10) [20]. Some of them show a high tissue specific expression like HspB9 and HspB10 which exist exclusively in testis or αA-crystallin (HspB4), which is primarily expressed in the eye lens (Figure 1), whereas others are ubiquitously present like Hsp27 (HspB1) and αB-crystallin (HspB5) [21,22,23,24,25]. Even though expression levels of sHsps are regulated precisely, co-existence of multiple sHsps in the same cell was observed and hetero-oligomeric assemblies of different sHsps seem to be a common feature [25,26,27,28,29]. One of the most prominent tissues with simultaneous sHsp expression is the eye lens where αA-and αB-crystallin (further referred as αA and αB, respectively) exist mainly as hetero-oligomers in a 3:1 ratio termed αL-crystallin [10,30,31,32]. The eye lens is remarkable in many respects. Ordinary tissue contains about 95% water with 5% protein content, leading to an overall protein concentration of maximum 65 mg/mL, whereas lens tissue has a reduced water content of not more than 70%,resulting in an increased protein concentration of up to 450 mg/mL [33,34]. The major protein family in lens tissue are the crystallins including α-, β-, and γ-crystallins, and representing 90% of the total protein amount [35,36]. At this high concentration, attracting and repelling forces between all crystallin molecules within the lens are of high importance to circumvent random fluctuations of the refractive index and reduce light scattering. The balance of protein interactions is therefore precisely controlled by the α-crystallins [35,37,38,39,40,41,42]. Furthermore, the transparent characteristic of the lens and the associated absence of any cell compartments implicate a drastic reduction in protein turnover, forcing its proteome to extend its lifetime. Thus, posttranslational and environmental modifications, like glycation of the proteins [43], might accumulate over time and change the behavior of many proteins. Due to limited protein turnover, damaged and aggregation-prone proteins accumulate over a lifetime and cataracts can be developed in an age-related fashion or by malfunction of specific members of the crystallin protein family. In this context, several point mutations in α-crystallins have been reported to result in hereditary cataracts formation, most prominent are the αA-R116C and αB-R120G variants [44,45]. In order to maintain lens transparency, the most common current model envisions sHsps as general aggregation suppressors via their characteristic chaperone function [46,47]. To date, it is not clear if a release mechanism from these sHsp-substrate complexes by ATP-dependent chaperones exists in the metabolically inactive lens [43]. However as described above, besides their chaperone function, α-crystallins additionally contribute to the crystalline interaction network of the lens.

In other non-lens tissues, failure in aggregation suppression by α-crystallins and other sHsps seems to correlate with several diseases, including neurological disorders, myopathies, multiple sclerosis, and different cancers. Specifically, sHsps are implicated in protein deposit diseases such Alzheimer‘s or Parkinson’s disease [25,48,49,50]. Additionally, α-crystallins were shown to play a divergent role in cancer development as αA can act as tumor suppressor inhibiting cell transformation, whereas overexpression of αB was reported in different tumor tissues, indicating an enhancing effect on tumor development [10,25]. Commonly, sHsps are not thought to be causative for the respective diseases, as long as no mutated variants of sHsp are present in the cells [25,33,37,44,51]. From a therapeutic point of view especially, α-crystallins have been investigated as potential drug targets. Lanosterol and oxysterol have been recently identified as interactors that seem to be able to reverse the aggregation of αA- and αB-crystallin and recover transparency of the lens in animal models [52,53].

## 2. The Structure of Human sHsps

**Figure 2:**
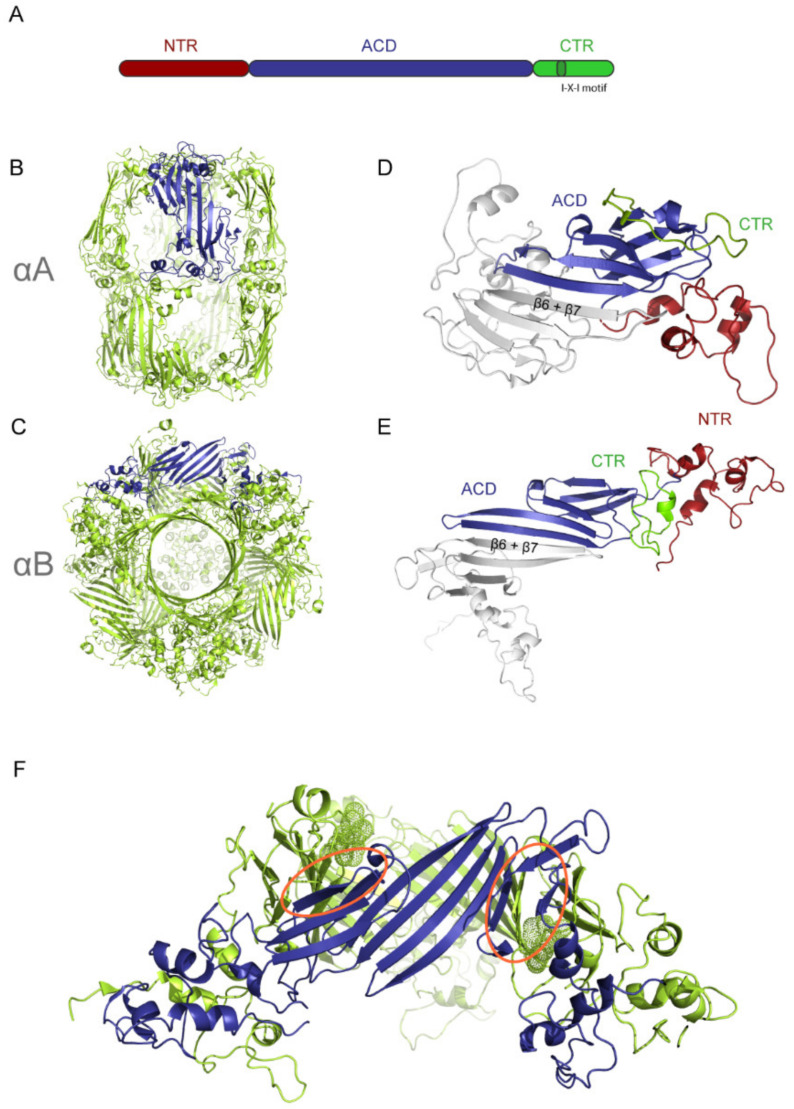
Primary structure of sHsps and structural comparison of αA and αB. Scheme of the common domain organization of a sHsps (**A**). Pseudoatomic models of (**B**) αA (PDB: 6T1R) and (**C**) αB (PDB: 2YGD). Both oligomers are colored in light green except one dimer which is highlighted in blue, respectively. A barrel-like shape is formed by 16 αA molecules with a D4-symmetry. In contrast, αB forms a hollow sphere with a tetrahedral symmetry composed of 24 molecules. For a more detailed comparison of (**D**) αA and (**E**) αB on the dimeric level, a dimer was extracted from each pseudoatomic model. The N-terminal region (NTR) (red), α-crystallin domain (ACD) (blue) and C-terminal region (CTR) (green) were highlighted for one of the dimers. Both NTRs have three helices, albeit they show different orientations [79,80]. (**F**) Hexameric subunit extracted from the pseudoatomic model of αB (PDB: 2YGD). The interacting IXI motifs are highlighted as dots. The β4–β8 groves are marked by orange ovals.

The most interesting feature of sHsps is their dynamic quaternary structure which allows them to form ensembles of oligomers with varying numbers of subunits. Typically, these ensembles show a broad distribution of oligomers around main species of 12–32 mers. Weak and dynamic inter-subunit contacts of all three sequence parts of the sHsps (NTR, ACD and CTR) are involved in oligomer assembly [58]. Homodimers formed by the ACDs of two protomers represent the basic building block of the higher order sHsp structures (Figure 2). The name giving ACD (Hsp20 domain, PF00011) with its 90–100 amino acids forms an immunoglobulin like beta sandwich structure. In mammals and other non-plant higher eukaryotes, the extended β6 + β7 sheets thereby form the dimer interface in an anti-parallel orientation (“β7-interface dimer”, [3]) (Figure 2D,E) [59,60,61,62,63,64,65]. This interface contact seems to be rather weak, a finding supported by the low observed dissociation constants and the recently observed dynamic behavior and monomerization of the ACD upon acidification [66,67]. The observation of odd numbers of subunits in mammalian sHsps [68,69,70,71,72] is additionally supporting the lability of the β7-interface. In contrast, sHsps from single cell organisms and plants, show a distinct β6-sheet located in an extended loop which mediates the dimer interactions by reciprocal swapping into an opposite monomer (“β6-swapped dimer”) [3,73,74,75,76,77,78].

The ACD is surrounded by the less conserved, flexible NTR and CTR. The latter is typically not longer than 20 amino acids, enriched in polar amino acids and solvent exposed to stabilize the protein in aqueous solutions. Furthermore, the CTR contains a conserved IXI/V motif (αA ^159^IPV^161^ and αB ^159^IPI^161^) [81] interacting with the hydrophobic β4/β8 groove within the ACD of a neighboring protomer [73,74]. Thereby, the IXI/V motif is surrounded by a palindromic sequence that enables the bi-directional binding to the β4/β8 groove [60,82,83]. Interestingly, the strength and flexibility of this interaction seems to vary even between closely related sHsps. For instance from NMR experiments, the αA CTR (which includes the IPV motif) was detected, whereas the CTR of αB crystallin was less resolved, thus demonstrating its higher flexibility [84]. Additionally, binding rates of the IXI/V motif to the ACD turned out to be the rate limiting step in subunit exchange kinetics [85].

In contrast to the CTR, the NTR is enriched in hydrophobic residues (Phe, Trp) [19]. Specifically, phenylalanines have been reported to be of importance for oligomer assembly and substrate binding [75,86,87]. Basic amino acids like arginines and prolines are also over-represented in the NTR, suggesting their involvement in oligomer assembly by mediating cation–π interactions with phenylalanine residues [88]. Additionally, the presence of arginine stabilizes proteins in solution by its chaotropic side chain [89], and as such, a contribution of the arginines to the chaperone mechanism was hypothesized [90]. As the NTR seems to be involved in substrate recognition [72,91,92,93,94,95,96,97], the low homology of the NTRs of sHsps from the same species points towards an influence of the NTR on the specificity of different sHsps. However, a significant overlap in the substrate spectra of Hsp27 and αB was described [98]. The NTR is highly flexible and seemingly intrinsically disordered at the very end of the N-terminus, as indicated by its susceptibility to proteolysis [99,100,101] and high hydrogen/deuterium exchange rates [79,82,91,102]. Nevertheless, some secondary structure elements in the NTR have been proposed, e.g., two helices for Hsp27 and three helices for αB and αA [79,80,82,103,104]. Additionally, ordered regions have been observed in the NTR of Hsp20 (HSPB6) when it is in complexes with a substrate protein [63], as well as in the crystal structure of the HspB2/HspB3 hetero-oligomer [64]. Interestingly, as visible in intrinsic order profiles, αA and αB seem to have overall the most ordered NTRs [49]. Some NTRs also contain a second IXI/V-like motif (e.g., Hsp20/HspB6) that was shown to also interact with the β4/β8 groove within the ACD [63,105]. Similarly, other sequence patches of the NTR were shown to bind to different grooves of the ACD, leading to varying quasi ordered states further highlighting the dynamics and structural heterogeneity of sHsps [64,82]. Some of these motifs are conserved and seem to control hetero-oligomerization [90,106]. Many posttranslational modifications have been reported for the NTR as well. The best characterized is the phosphorylation of αB and Hsp27, which seems also to influence the respective contacts of the NTR in oligomer formation [29,107,108,109,110,111,112,113,114].

The described flexibility and plasticity of their NTRs and CTRs together with the dynamic properties of oligomers, render structural analysis of specifically human sHsps highly challenging. Therefore, only a limited number of atomic structures for isolated ACDs or truncated variants of human sHsps are available, and our understanding of the full-length, oligomeric proteins is still highly limited. Together with a very low number of high resolution structures of sHsps from other organisms, the current view is that sHsp oligomers are assembled hierarchically involving the contacts of all three sequence parts [58]. In the following, we describe in a comparative manner the current understanding on the structure of αA and αB, which represent the only two oligomeric human sHsps which have been described at pseudo-atomic resolution until now.

Interestingly, these pseudo-atomic models of recombinantly expressed αA and αB, generated by hybrid approaches combining structural data from cryo-electron microscopy (cryo-EM), NMR spectroscopy, small angle X-ray scattering (SAXS), and crosslinking-mass spectrometry (crosslinking-MS) together with molecular modelling simulations, indicate that the oligomers of the two sHsps are quite different in symmetry, dynamics and assembly organization [79,80,85,104].

### 2.1. Structural Comparison of αA and αB

For αB, currently two major 3D models are under discussion: one majorly based on cryo-EM data and the other on NMR data [58,80,100,104,115,116,117]. Both models describe a football like 24 mer in pseudo-atomic resolution (Figure 2). The particles forming hollow spheres of ~13.5 nm diameter with tetrahedral symmetry and a hierarchical assembly principle is common to both oligomeric models. The first level of hierarchy is represented by a dimer formation mediated by the elongated β6 + β7 sheets in a head-to-tail orientation. The cryo-EM based model (9.4 Å; with the 24 mer representing ~30% of all detected particles), which was further refined with the help of crosslinking-MS, NMR and molecular modeling, postulates two slightly different conformers of dimers participating in the oligomer assembly [80]. In one conformer, the ACD and NTR are bent by an angle of 55 ° setting its NTR and CTR as opposite ends (“bent monomer”). In the second conformer ACD and NTR are positioned almost linearly (“extended monomer”). This assumption represents the only way in which dimers could be integrated in high resolution cryo-EM electron densities. In contrast, the NMR-based model (refined with small angle X-ray scattering (SAXS), refers to only one conformation of all monomers and dimers in agreement with the single set of NMR resonance observed for these regions [58,104,117]. The second level of hierarchy in both models involves the C-terminal IXI/V motif that mediates hexamer formation of three dimers by binding to the β4/β8 groove of the neighboring dimer (Figure 2F). The third level of hierarchy is the association of hexamers to 24 mers mediated by the contacts of the NTR. Deletion mutants of the NTR, as well as phosphorylation in the N-terminal region (which leads to disassembly of the oligomers) are in good agreement with the models [100,104,118]. Thus, all three sequence parts of the sHsps contribute to oligomer assembly. Modulation on one or up to all three respective regions can change the stability of the oligomers and the overall ensemble composition by addition or release of building blocks. Further PTMs, like phosphorylation of the NTR, represent a trigger for a structural switch, destabilizing the contacts of the NTR followed by the disassembling of the oligomers [29,100,113,119]. Similarly, acidification seems to trigger structural rearrangements of the ACD. Investigations of a conserved histidine (His104) located within the dimer interface revealed the respective structural changes upon acidification by modulating the interface stability [65,120].

Recently, a pseudo-atomic 3D model of αA-crystallin (9.0–9.8 Å) in its reduced state was proposed [79]. This model is also based on cryo-EM data, refined by crosslinking-MS, NMR, and molecular modeling. The assembly shows barrel-like architectures with a 6–16 nm width and 13–14 nm length (Figure 2B). The most abundant particles were assigned to 12 mers (36%; D3-symmetry), 16 mers (27%; D4-symmetry) and 20 mers (19%; D5-symmetry). In contrast to the αB models, the building block of the oligomers is a z-shaped tetramer consisting of two dimers. Tetramer formation is mediated by inter-dimer interactions via NTR contacts across the equator (‘equatorial N-terminal interface’), whereas apical NTR interactions between the tetramers (‘apical N-terminal interface’) are responsible for oligomer formation. Thus, the CTR seems not to be critical in the formation of the tetrameric building block or higher oligomers, but it still supports oligomerization by binding of the IXI/V-motif to the neighboring protomer.

The equatorial plane of the barrel differs between 12 mers/16 mers and 20 mers, as neighboring tetramers are not connected in 12 and 16 mers but linked via a well-resolved density bridge in the 20 mers assemblies.

Most likely, this density results from the intermolecular binding of the IPV motif into the β4/β8 pocket of an adjacent protomer ACD (3D domain-swapped configuration), while in case of lacking density in the equatorial plane the IPV motif binds intramolecularly into the β4/β8 pocket of the ACD of the same polypeptide chain (non-3D domain-swapped configuration). Furthermore, paramagnetic relaxation enhancement (PRE) experiments confirmed that the 3D domain-swapped conformation is scarce (<20%) in the oligomeric assembly. Similar observations of swapped and non-swapped CTRs were reported for crystal structures of different truncated variants of bovine and zebrafish αA [60]. Secondary structure prediction revealed three helices within the NTR which are connected by flexible loops highly similar to those observed in the cryo-EM based αB model.

One major difference between human αA and αB is the redox sensitivity of αA, driven by its two cysteine residues located at the C-terminal end of the ACD, which can form an intramolecular disulfide bond in vivo [121,122,123,124,125,126,127]. Note: not all mammalian αA-crystallins contain two cysteines and are redox sensitive but the two cysteines are conserved in long-lived species like primates. The relevance of the redox state for αA is highlighted by the observation that the addition of another cysteine, such as the R116C mutation, induces cataracts and leads to drastic changes in structure and activity of αA [128]. When the structure of oxidized αA was analyzed with the cryo-EM based hybrid approach, more polydisperse and larger oligomers (17.7 nm in length) were observed. A preliminary 3D reconstruction of a 32 mers showed the same organization principle for this oligomer assembled from z-shaped building blocks of tetramers (dimer of dimers) [79]. It seems like the particles expand via stepwise integration of further building blocks. As shown for Sip1 from *Caenorhabditis elegans*, which forms structurally similar barrel-shaped oligomers as well, such expansion and reduction of the building blocks of the oligomer are most likely key to the correlation with changes in activity as explained below [62]. Oxidation introduces an intramolecular disulfide bridge which represents a cross-strand disulfide. Such disulfides are commonly thought of as reactive redox-based conformational switches which lower the stability of the overall structure as they introduce tension by the conformational distortions introduced by the disulfide bond.

From the eye lens, native αA and αB were isolated as potentially hetero-oligomeric complexes (αL-crystallin, αL) in a stable 3:1 ratio [30]. Later, it was shown that the composition of the hetero-oligomers in fetal lenses is 2:1, whereas in aged lenses a 3:2 ratio was reported [31]. Furthermore, the expression levels of αA and αB change independently during differentiation of bovine lens epithelia cells to fiber cells (Figure 1B) [57]. In epithelial cells, αA levels are low and expression is enhanced during differentiation while αB levels decrease during differentiation [57,129,130]. In the light of the recent structural observations, it needs to be re-addressed whether the co-isolation of αA and αB crystallin during extraction from the lens can indeed be interpreted as the formation of hetero-oligomers. At least the current structural models do not hint to a clear picture of a hetero-oligomer. As building blocks and the usage of contacts seem to be different, it is hard to imagine what an αL oligomer might look like.

The complexity of αL is even higher. The size distribution of the extracted αL hetero-oligomers is more heterogeneous including oligomers from 300 to 1000 kDa [131,132,133]. As for the homo-oligomeric complexes, the size distribution of αL can be shifted by temperature, concentration, pH, ionic strength and lens age [22]. The broad range of size heterogeneity may be further influenced by post-translational modifications [30].

By using crosslinking-MS, a high similarity of the interaction sites in αA, αB and αL oligomers from bovine fetal lenses was proposed. More precisely, the NTR was observed to interact with the neighboring subunit most likely in the region of the dimer interface within the ACD, pointing also to a high flexibility of the NTR [134]. Applying an in vitro peptide scan approach with recombinant purified proteins, specific regions in the NTR (amino acids 42–57 and 60–71) and in the ACD (amino acids 88–123) of αB were determined to interact with αA during hetero-oligomerization [135]. The most recent report on the structure of αL extracted from a bovine eye lens proposed a model with a resolution limit of 2 nm by using negative stain electron microscopy (EM), dynamic light scattering (DLS), and analytical ultra centrifugation (AUC). An asymmetrical bean-like shape of 13 × 19 nm with a dense core and filamentous “kernel” but without a central cavity was described as the most abundant particle. The molecular weight was narrowed down to 750–830 kDa equaling 35–41 mers. In agreement with previous data such αL oligomers are notably larger than the respective homo-oligomeric particles of recombinant proteins. From 18,000 heterogenic, selected particles a low resolution 3D model based on a 2.4:1 ratio of αA:αB was reconstructed [136]. However, it remains inconclusive how the two proteins are arranged within oligomers. Additionally, it remains unclear if the ensemble is composed solely of hetero-oligomers or if homo-oligomers of the two crystallins are also included.

In vitro reconstituted hetero-oligomers from recombinant proteins (commonly named αAB) have on average lower molecular masses compared to extracted αL oligomers from lenses [137]. Still they show a higher density with increased molecular weight compared to in vitro homo-oligomers of αA or αB [138,139]. In this context, the different conditions in the eye lens compared to the conditions used for in vitro reconstitution and characterization need to be considered. In the lens, protein concentrations of 100–400 mg/mL generate a highly crowded milieu, whereas structural investigations in vitro are often performed in low µM ranges [140,141]. Recently, in a comparative approach for αB, a decrease in the oligomer size from 40.7 S in crowding agents to 10.7 S in diluted solution has been reported [142]. In contrast, results for bovine αL at different concentrations reported only slight concentration dependent size changes between 5–10 nm in SAXS experiments [143], or an average diameter of 15 nm at concentrations up to 290 mg/mL [144].

**Figure 3:**
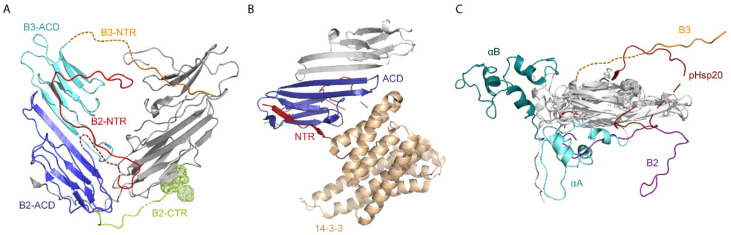
Complex formation and conformational diversity of NTRs. (**A**) Tetrameric assembly shows HspB2 and HspB3 in a 3:1 ratio (PDB: 6F2R) [64]. The crystal structure consists of two dimers. One built of two HspB2s (light grey, homodimer) and the other of HspB2 and HspB3 (heterodimer). The second one is formed by an interaction of the β7-interface with their ACDs (B3–ACD: cyan and B2–ACD: blue). This dimeric structure is stabilized by an interaction of the HspB2–NTR (red, dashed lines are modeled) with the HspB3–ACD. Further stabilization is achieved by interactions with the neighboring homodimer via HspB3–NTR (orange, dashed lines modeled) and HspB2–CTR (light green, V-Y-I motif is highlighted with spheres). Short peptides which had not been assigned to any sHsps within the structure are excluded from visualization. (**B**) Part of the hetero-tetrameric complex (PDB: 5LTW) of phosphorylated Hsp20 (pHsp20) with 14-3-3 (wheat colored, only one of the two dimers is shown) [63]. Both proteins are known to build stable complexes together. Two unique interactions were formed: First pHsp20–NTR (red) interacts with the binding groove of 14-3-3 and second the pHsp20 ACD dimer interacts with one 14-3-3 protein. (**C**) Visualization of NTRs from five human sHsps confirms their different three-dimensional conformations. HspB2 (NTR: purple) and HspB3 (NTR: orange, dashed line modeled) were extracted from the X-ray structure of its hetero-oligomer (PDB: 6F2R) [64], as well as pHsp20 (NTR: red) from the sHsp–substrate complex (PDB: 5LTW) [63]. αA (NTR: cyan) and αB (NTR: petrol blue) are extracted from the two pseudoatomic models (PDB: 6T1R and 2YGD) [79,80]. All five monomeric structures are superimposed by their ACDs. To highlight the structural variability of the NTRs, ACDs and CTRs are colored in light grey.

Taken together, the recent structural studies represent a tremendous step forward in the understanding of α-crystallins. It needs to be emphasized that such highly heterogeneous and polydisperse protein ensembles are extremely challenging to reconstitute and interpret. It will be fascinating to see if future studies can solve the conundrum of how αL can assemble from αA and αB. The available structures indicate that the key to overall structural flexibility might be envisioned in the variability of the contacts formed by three sequence parts (NTR, ACD, CTR). This is further supported by progress in understanding the structure functional relationship of some other human sHsps (Figure 3) [63,64,66,145].

### 2.2. Structures of Other Human sHsps

Recently, a crystal structure for a tetrameric assembly of human HspB2 and HspB3 was presented (Figure 3A) [64]. Typically, HspB2 and HspB3 are found as hetero-oligomers in neuromuscular cells. In the crystallized HspB2–HspB3 tetramer the proteins are present in a 3:1 ratio forming a dimer of dimers, including a homodimer of HspB2 and a HspB2–HspB3 heterodimer in antiparallel orientation. All four ACDs form a clamp-like, flattened tetrahedron whereby the dimers are interconnected via the IXI/V motifs of the CTRs reaching to the ACDs of the neighboring HpsB2 subunit at the tip of the structure. As the antiparallel orientation bends the dimers away from each other, the elongated CTR of HspB3 forms an additional stabilizing contact with the adjacent ACD of the neighboring HspB2 homodimer. Unfortunately, the NTRs are not completely resolved which indicates that they might be at least partially flexible. The resolved parts of the NTR form additional contacts with grooves on the ACDs of the dimeric building block. Interestingly, the observed contacts indicate that the homo-tetramer of HspB2 would not be stable, which might be the reason for the formation of the HspB2–HspB3 hetero-oligomer in vivo. HspB3 on its own however, is able to form heterogeneous oligomers [98]. Both proteins also show chaperone activity on their own in vitro.

Moreover, the crystal structure of Hsp20 (HspB6) in complex with the phosphoproteome regulator protein 14-3-3 was resolved (Figure 3B) [63]. Upon phosphorylation, dimeric pHsp20 specifically interacts with dimeric 14-3-3, thereby presumably acting as displacer for other regulatory proteins from 14-3-3 complexes and thus modulating the interaction network of 14-3-3 and the phosphoproteome. In the crystallized hetero-tetrameric complex, dimeric pHsp20 (phosphorylated Hsp20) binds with its ACDs to one of the two subunits of the 14-3-3 dimer (see [63] for a scheme of the complex). Hsp20 lacks the CTR and was demonstrated to be a homodimer on its own, thus contacts of the CTR are not involved. However, the asymmetric pHsp20/14-3-3 complex is further stabilized by contacts of the NTRs of the pHsp20 subunits (residues 13-RRApSAPLP-20; including phosphoserine 16) [63], which become structured upon binding to grooves on 14-3-3 (Figure 3B). Additionally, a conserved sequence stretch of one of the Hsp20 NTRs (residues 27-RLFDQRFG-34; [63,90]) binds to a groove formed on the ACD dimer (β3/β3), which represents a similar structural arrangement as observed for the NTR-ACD contacts in the HspB2-HspB3 hetero-oligomer [64]. Interestingly, the two NTRs of pHsp20 display different arrangements, again highlighting the structural flexibility modulating the contacts formed and once more defining the NTR as key player for structural variability (Figure 3C) [17,82,145]. Several grooves on the ACD additionally provide the necessary, suitable contact sides for the NTR, presumably allowing the variability in oligomer formation [64,145].

## 3. Chaperone Function of Human sHsps

As mentioned briefly above, sHsps prevent partially unfolded proteins from irreversible aggregation by ATP-independent stabilization. This promiscuous stabilization of aggregation-prone proteins in the cellular context is of high importance in multiple respects: (1) to avoid exposure of hydrophobic surfaces that can mediate unfolding and aggregation of further proteins and lead to loss of protein function on a much broader scale; (2) to protect folding intermediates from proteolysis and hydrolysis; (3) to enable refolding from the resulting stable sHsp-substrate complexes and conserve energy, as synthesis of new proteins is particularly restricted for the lens crystallins (4) to maintain lens transparency which is most likely correlated to (1). To fulfil these functions, the presence of sHsps during unfolding of substrate proteins is mandatory. Already aggregated protein deposits cannot be rearranged by sHsps retrospectively [146,147]. Therefore, sHsps are typically present at high levels in cells under physiological conditions and are further over expressed upon diverse stress situations [3,26,148,149].

In vitro sHsps were demonstrated to have a chaperone function in terms of the suppression of thermal, chemical or UV induced unfolding of many model substrates [3,150,151,152,153,154].

Different binding modes of sHsps to substrates possibly exist, as in vitro studies showed that the formation of stable, soluble sHsp–substrate complexes or alternatively, aggregate-like complexes with incorporated sHsps (which might be predominant in vivo) is dependent on the substrate used and the ratio of sHsp to substrate [8,9,12,155,156,157]. Additionally, transient binding of early unfolding intermediates was observed for some sHsps [146,147,153]. Low affinity binding of substrates was postulated especially for the ACD mediated interactions with amyloids, whereas amorphous aggregates are bound stably via the NTR [158]. Thus, binding affinity might be dependent on the sequence region of the sHsp which is involved in substrate recognition.

The rather old observation that sHsps are incorporated into cellular aggregates e.g., inclusion bodies in bacteria [9], has become highly interesting in recent years as it could be interpreted as a so called “aggregase” or “sequestrase” function for some Hsps. Specifically, for a sHsp from baker’s yeast (Hsp42), it was demonstrated that it enhances the aggregation of some substrate proteins [159,160,161,162]. Hsp42 induces the sequestration of the respective misfolded proteins into cytosolic CytoQ aggregates as a kind of protein quality control strategy. It remains to be seen if other, and especially some of the ten human sHsps show such a “sequestrase” activity as well, and if such a specialization might be an explanation for the high number of different sHsps encoded in human and other higher eukaryotes [19,20].

Refolding of proteins from sHsp-substrate complexes as well as from sHsp-incorporating aggregates can be conducted by ATP-dependent chaperones. In mammalian cells, the recovery of substrate proteins is mediated by the Hsp70/Hsp40 chaperone system [11,163,164]. For some sHsps like Hsp22 (HspB8) the interaction between sHsps and Hsp40/Hsp70 is assumed to be facilitated by the Hsp70 co-chaperone Bag3 [165,166,167,168,169]. Additionally, some sHsps interact as well with proteins in their native conformation like pHsp20 (phosphorylated HspB6) with 14-3-3 (see above) or Hsp27 (HspB1) and αB with cytoskeleton components like actin [63,111,170,171,172,173,174]. Such interactions might be the basis for the involvement of sHsps in the regulation of cytoskeleton dynamics or the redox state of cells as in their presence the level of reactive oxygen species (ROS) was reported to decrease. However, from a mechanistic point of view such regulatory implications of sHsps in cellular processes are not understood up to now.

**Figure 4:**
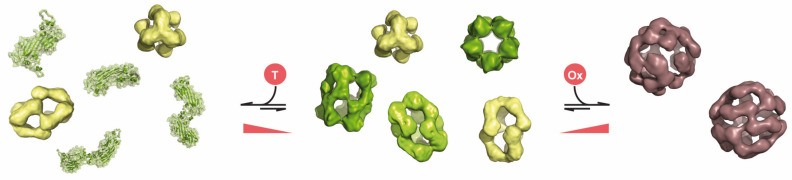
Scheme of αA activation at physiological conditions αA consists of an ensemble of oligomers where 12–20 mers are predominant (middle panel) [79]. Stress conditions like increased temperature (T) presumably lead to a shift of the ensemble composition to a smaller species [138,152,183,189]. Most likely tetrameric and dimeric building blocks dissociate from the oligomers which leads to an increase in the chaperone activity of αA (left panel). Oxidative stress (Ox; or more generally redox-stress) leads to the enrichment of larger oligomers (predominantly 32 mers; right panel, dark coral colored) [79]. In these larger oligomers intra-molecular disulfide bonds are enriched, destabilizing the particles and increasing their dynamics. This also leads to activation of the chaperone activity of αA as subunits or building blocks can be presumably detached more easily from these oxidized complexes. Oligomeric structures were generated with the following PDB and electron microscopy data bank (EMDB) files: 6T1R, EMD-4895, EMD-4894, EMD-4896 [79]. Furthermore, an EM density map of αA_ox_ was kindly provided by Dr. Carsten Peters and Dr. Christoph Kaiser (Technische Universität München).

The main protein components of the eye lens are α-, β- and γ-crystallins. The characteristic of this protein family is the content of two domains compromising two consecutive Greek key motifs that fold together in two beta-sheets [35]. The α-crystallins demonstrate chaperoning properties towards β- and γ-crystallins but also most likely towards themselves [175,176], as stability investigations have shown that γ-crystallin is more stable than α- and β-crystallins [177]. Thus, it is expected that α-crystallin is present in a mixture of native and denatured conformation in the eye lens [37]. By binding destabilized proteins, the α-crystallins are thought to maintain lens transparency according to common understanding of the sHsp chaperone mechanism (Figure 4). As long as the substrate–sHsp complexes do not exceed the size of soluble, not light-scattering complexes, transparency is ensured and resistance against oxidative-stress (and other stressors) as well as thermo-tolerance is conferred [178,179]. In this context it is intriguing that until recently it was unclear if αA shows indeed comparable mechanistical properties to αB [32]. More specifically, the link between the ensemble dynamics and the observed aggregation suppression properties is not yet understood in detail for αA. sHsps are able to recognize their substrates by switching between states of low and high affinity. Under physiological conditions, the low affinity state is predominant, where defined triggers (e.g., stress situations) lead to a shift to the high affinity state and thus, activate the chaperone activity of the respective sHsp. This shift between affinity states is regulated by intrinsic structural dynamics and commonly correlates with the composition of oligomeric species within the ensemble (Figure 4) [100,180,181]. Typically, a shift to a higher content of smaller species (often dimers) leads to activation of sHsps [181]. Various triggers like the presence of unfolded substrates, changes in temperature and pH, phosphorylation or posttranslational modifications, as well as hetero-oligomer formations [17,26,28,62,100,153] have been described. Interestingly, many of these triggers are still not understood in detail. A comparative analysis of hetero-oligomer formation of the tripartite system of Hsp27 (HspB1), αB and Hsp20 (HspB6), which represent the group of sHsps present simultaneously in many tissues [25,111], showed that the effect of hetero-oligomer formation on the composition of the sHsp ensembles and their chaperone activities depends strongly on the respective sHsps involved [26]. Hetero-oligomer formation between Hsp27 and αB leads to an ensemble which is enhanced in species larger than the individual homo-oligomers. In contrast, the interaction of dimeric Hsp20 (HspB6) [26,27] with either Hsp27 or αB oligomers enriches the ensemble with smaller oligomers. While the larger Hsp27-αB hetero-oligomers are less active, Hsp20 (HspB6) activates αB by oligomer dissociation. The chaperone activity of Hsp27–Hsp20 hetero-oligomers however, is modulated in a substrate-specific manner, most likely by specifically enriching a Hsp27–Hsp20 heterodimer. These heterodimeric species seem to allow tuning of the chaperone properties towards certain substrates. Interestingly, recent evidence showed that hetero-oligomerization is controlled by conserved motifs within the NTR [106]. As the NTR is also involved in substrate recognition and controls the binding to certain substrates [182], it might be hypothesized that the respective tuning mechanism of the chaperone activities by hetero-oligomerization relies on structural switches in the contacts of the NTR resulting in changes of the accessibility of certain motifs or sequence parts.

Recently for αA, the presence of a splice variant with truncated NTR, which has been observed in preparations of cataract eye lenses [33], was described as a new type of trigger as well [103]. This splice variant shows the characteristic α-crystallin secondary structure, and it exists on its own predominantly in a monomer–dimer equilibrium and displays only low chaperone activity. However, the variant is able to integrate into higher-order oligomers of canonical αA- and αB-crystallin as well as their hetero-oligomer. The presence of the variant leads to the formation of new types of higher-order hetero-oligomers with an overall decreased number of subunits and enhanced chaperone activity. Thus, alternative mRNA splicing of αA represents a further regulatory trigger and it remains to be verified if such a trigger also exists for other (human) sHsps.

As described above, the structural analysis of αA revealed that the design principle is similar to other sHsps and the regulation might work in a comparable manner (Figure 2 and Figure 4). It should be noted that the active substrate binding species of αA is still unknown, but dimers are most likely candidates for this, as well as for other sHsps [183]. Additionally, αA seems to be triggered also by oxidation (Figure 4). In young lenses, especially oxidized αA (αA_ox_) are highly abundant [184], and it seems likely that in the eye lens αA switches between different redox states. Thereby the redox potential of the intramolecular disulfide bridge in human αA is similar to that of thiol-disulfide oxidoreductases [79,185]. Interestingly, oxidation correlates with an increase in chaperone activity. αA_ox_ seems even to be able to transfer its intramolecular disulfide to destabilized substrates and might contribute as an integral component to lenticular redox homeostasis. Thus, in terms of regulation, oxidation represents a further new activation mechanism for αA, and it needs to be clarified if other sHsps are regulated in a similar fashion. Hsp27 can protect cells and tissues against oxidative stress. Structure rearrangements induced by oxidation followed by changes in function have been described in the past but are not understood in detail yet, as structural data on the Hsp27 oligomer are still missing [186,187,188].

However, oxidation of αA leads to an enrichment of larger oligomers which is on first sight contradictory to the common assumption that activation of sHsps is accompanied with dissociation into smaller species. However it needs to be noted that αA_ox_ shows higher polydispersity, enhanced dynamics and seems to be destabilized. Thus, it might well be that within the ensemble the active substrate binding species, which are most likely dimers or tetramers, are still enriched or more likely to dissociate from the oligomers.

## 4. Conclusions

Follow-up studies on the observations from the early 1900s have led to a consensus model on the function of (human) sHsp which is closely linked to their structural key feature: the formation of variable, dynamic oligomers. Recent progress in resolving the structures of oligomeric complexes of human sHsps allows hypothesizing on the basic principles of the assembly and gives first insights into their tremendous conformational flexibility. This is of special interest as the conformational variability might also be the key to understanding the functional modulation of these proteins by conformational switching.

The key points of the current understanding are:

- sHsps are an integral component of the chaperone network and help to sustain the solubility and functionality of the proteome upon stress
- Under physiological conditions they are involved in a number of regulatory processes within the cell
- They are implicated in a variety of diseases but are usually not the causative component
- Their function is closely correlated to their structural variability
- They are commonly organized in three sequence parts, a conserved ACD flanked by divergent NTRs and CTRs
- Most sHsps form dynamic ensembles of oligomers with a variable number of subunits; some sHsps even form hetero-oligomeric species
- The assembly processes are controlled by the three sequence parts which form contacts in a hierarchical manner
- The conformational flexibility of the NTR, together with a set of binding groves on the ACD, are most likely the key to the variable oligomeric assemblies
- Changes in the composition of the dynamic ensembles of oligomers are linked to their chaperone activity and the recognition of substrates
- Changes in dynamics and composition of the ensembles lead also to modulated substrate specificities

Nevertheless, while the basic and common principles seem to become less nebulous, a lot of important questions need to be addressed (which is now possible). For example, the substrate range and the specificity of human sHsps in particular, are still not sufficiently defined despite progress in recent years. Specifically, comparative approaches under physiological versus different stress, tissue or developmental conditions are lacking. Such studies might be key to understanding the regulatory implications of sHsps and to clarify their potential as drug targets in various diseases. Additionally (and surprisingly), it still remains elusive how substrate recognition occurs mechanistically, e.g., which sequence parts (and maybe motifs) interact with the substrates, if these overlap with the sites involved in oligomer formation, and how substrate specificity is defined. Taken together we now seem to be at the dawn of a detailed mechanistic understanding of these still enigmatic molecular chaperones, as the availability of several, long-needed oligomeric structures allows addressing a variety of open issues.
